# Sensitive and rapid detection of *Candida tropicalis* using closed dumbbell-mediated isothermal amplification

**DOI:** 10.3389/fcimb.2026.1875246

**Published:** 2026-08-21

**Authors:** Yangbo Fu, Jing Shen, Haizhong Jiang, Yanli Zhang, Xuhan Chen, Rui Mao

**Affiliations:** 1 The First Affiliated Hospital of Ningbo University, Ningbo, Zhejiang, China; 2 Ningbo No. 2 Hospital, Wenzhou Medical University, Ningbo, Zhejiang, China; 3 Ningbo Institute of Life and Health Industry, University of Chinese Academy of Sciences, Ningbo, China

**Keywords:** *Candida tropicalis*, closed dumbbell mediated isothermal amplification (CDA), on-site visualization, real-time fluorescence CDA, sensitive and rapid detection

## Abstract

**Introduction:**

*Candida tropicalis* is responsible for a substantial proportion of invasive candidiasis cases and is recognized as the second most prevalent causative agent, only behind *Candida albicans*. A documented rise in invasive systemic infections and sepsis due to *C. tropicalis* has contributed to the increased mortality among vulnerable patient populations, highlighting the critical need for heightened clinical vigilance. Early detection of *C. tropicalis* is essential for guiding appropriate antifungal therapy and is associated with improved clinical outcomes.

**Methods:**

To address the need for rapid and reliable detection of *C. tropicalis*, this research aimed to develop a novel closed dumbbell-mediated isothermal amplification (CDA) assay characterized by high speed, specificity, and efficiency. A set of specific CDA primers targeting the conserved *ITS2* gene of *C. tropicalis* was successfully designed, selected, and validated, resulting in a simple and efficient detection method. The *C. tropicalis* CDA assay for *in vitro* diagnostic applications demonstrated robust performance, with its sensitivity, robustness, stability, and specificity systematically evaluated using 364 batches of diverse DNA samples.

**Results:**

The *C. tropicalis* CDA assay demonstrated perfect diagnostic performance with apparent 100% sensitivity (95%CI = 97.0%–100%), specificity (95%CI = 98.5%–100%), and accuracy (95%CI = 99.0%–100%) in a primary evaluation using 364 batches of diverse DNA samples, with a limit of detection of 5.6 × 10^−6^ ng/μl (approximately 10 copies/μl), which is not significantly different from that of real-time quantitative PCR (qPCR), indicating comparable analytical sensitivity.

**Discussion:**

Validated in both real-time fluorescence and endpoint visual formats, the *C. tropicalis* CDA assay demonstrated high specificity, sensitivity, and accuracy, along with robust stability and reproducibility, supporting its future application potential.

## 1 Introduction

*Candida* species represent the most prevalent opportunistic fungal pathogens and account for the majority of invasive fungal infections (Lopes and Lionakis, 2022). *Candida tropicalis* is responsible for a significant proportion of invasive candidiasis and is recognized as the second most prevalent *Candida* species, behind *Candida albicans*. Typically, *C. tropicalis* is capable of extensive proliferation following immune compromise or changes in the skin barrier, leading to local infection and potential systemic invasion. A well-documented increase in invasive systemic infections and sepsis attributable to *C. tropicalis* has been observed, particularly among vulnerable patient populations. This trend is associated with increased mortality and underscores a pressing need for enhanced diagnostic and therapeutic vigilance. Epidemiologically, *C. tropicalis* infection is characterized by enhanced virulence, easier progression to candidemia, higher mortality, and poorer prognosis relative to *C. albicans* infection (Liu et al., 2024; Yu et al., 2025). In addition, *C. tropicalis* is the second or third leading cause of invasive fungal infections in patients with cancer (Nucci and Colombo, 2007). Moreover, evidence indicates that *C. tropicalis* is becoming increasingly resistant to standard antifungal therapy (Deorukhkar et al., 2014). Therefore, rapid and precise identification of *C. tropicalis* is of critical importance for the prevention, management, and effective therapy of this opportunistic but clinically formidable pathogen.

It is anticipated that polymerase chain reaction (PCR) will remain indispensable for the molecular detection of pathogens in the years ahead (Zhu et al., 2020). However, the most frequently used quantitative PCR (qPCR) detection is constrained by its reliance on sophisticated and costly thermal cycling equipment, skilled operators, and stringent operational environments (Farrar and Wittwer, 2015). The pursuit of point-of-care (POC) pathogen detection has driven the development of isothermal amplification technologies, including loop-mediated isothermal amplification (LAMP), polymerase spiral reaction (PSR), and recombinase polymerase amplification (RPA), offering viable alternatives to conventional PCR-based assays (Jiang et al., 2016; Noguchi et al., 2017; Wang et al., 2021; Li et al., 2023). Among these methods, LAMP has been successfully commercialized and has demonstrated high specificity, simplicity, and sensitivity, allowing for the detection of targeted DNA within an hour (Noguchi et al., 2017; Lin et al., 2021; Ou et al., 2021; Mao et al., 2022b; Tu et al., 2024; Zhang et al., 2026). Nevertheless, the application of LAMP is constrained by its stringent primer design requirements and its propensity for nonspecific amplification (Gonçalves Dda et al., 2014; Mao et al., 2018; Gomez-Gutierrez and Goodwin, 2022). Accordingly, the adoption of an advanced isothermal nucleic acid amplification technique provides a promising pathway toward rapid and sensitive detection of *C. tropicalis*.

The newly developed closed dumbbell-mediated isothermal amplification (CDA) method is an optimization of LAMP, leveraging a unique single-stranded closed dumbbell DNA structure to enable rapid nucleic acid annealing and extension (Mao et al., 2022a). The CDA method is characterized by efficiency, high sensitivity, and high specificity, with demonstrated applications including the detection of *Rickettsia raoultii*, *Klebsiella pneumoniae*, *Vibrio parahaemolyticus*, and *Listeria monocytogenes* (Gui et al., 2022; Zhang et al., 2024a, 2024, 2025) and the identification of fish species (Jiang et al., 2025). CDA was established to enable rapid and simple detection of *C. albicans* (Zhang et al., 2025a).

To our knowledge, no prior study has applied CDA to *C. tropicalis* with high sensitivity and specificity. The internal transcribed spacer 2 (ITS2) region was selected as the conserved DNA target for *Candida* species identification, enabling rapid and specific DNA recognition (Ciardo et al., 2006). After sequence evaluation using the Basic Local Alignment Search Tool (BLAST) against the NCBI GenBank database, the targeted *C. tropicalis* DNA sequence was selected for the development of a CDA-based method enabling both gene identification and isothermal signal amplification. The specificity, sensitivity, and detection limit of the developed *C. tropicalis* CDA assay were evaluated and compared with those of real-time qPCR, a well-established reference method. Furthermore, diverse clinical DNA samples were used to assess the practicality and reliability of the assay. Collectively, the CDA assay represents a powerful diagnostic tool for the rapid and straightforward identification of *C. tropicalis* infections.

## 2 Materials and methods

### 2.1 Ethics approval statement

Ethical approval, including the waiver of informed consent of the clinical samples collected by our research team, was approved by the Ethics Committee of Clinical Investigation in the First Affiliated Hospital of Ningbo University (under approval number 2024-096A-02), which is consistent with our previous findings supported by the same project (Zhang et al., 2025a). The research conformed to the principles of the Helsinki Declaration. The study involved no more than minimal risk to subjects, and no personal information was obtained.

### 2.2 DNA samples of different origins

A standard strain of *C. tropicalis* (BNCC 186815) was obtained from the BeNa culture Collection (BNCC). A total of 26 clinical DNA samples of *C. tropicalis*, four of *C. albicans*, four of *Candida glabrata*, three of *Candida parapsilosis*, three of *Pseudomonas aeruginosa*, four of *Streptococcus pneumoniae*, three of *Stenotrophomonas maltophilia*, one of *Aspergillus fumigatus*, four of *Haemophilus influenzae*, three of *Streptococcus agalactiae*, three of *Escherichia coli*, three of *Shigella sonnei*, four of *Staphylococcus aureus*, three of *Vibrio Parahaemolyticus*, and four of *K. pneumoniae* were collected from July 2024 to November 2024 by the research team from the Laboratory of the First Affiliated Hospital of Ningbo University. In practice, alveolar lavage fluids of different origins were collected and extracted to obtain DNA samples. Clinical DNA samples of *C. tropicalis* and non-*C. tropicalis* were identified using next-generation sequencing (NGS) and subsequently adopted for the assessment of the method developed in this study. DNA samples were extracted using DNA purification kits (Sangon, Shanghai, China) and stored at −20°C.

### 2.3 CDA primer design and selection targeting of *C. tropicalis* DNA

**Figure 1:**
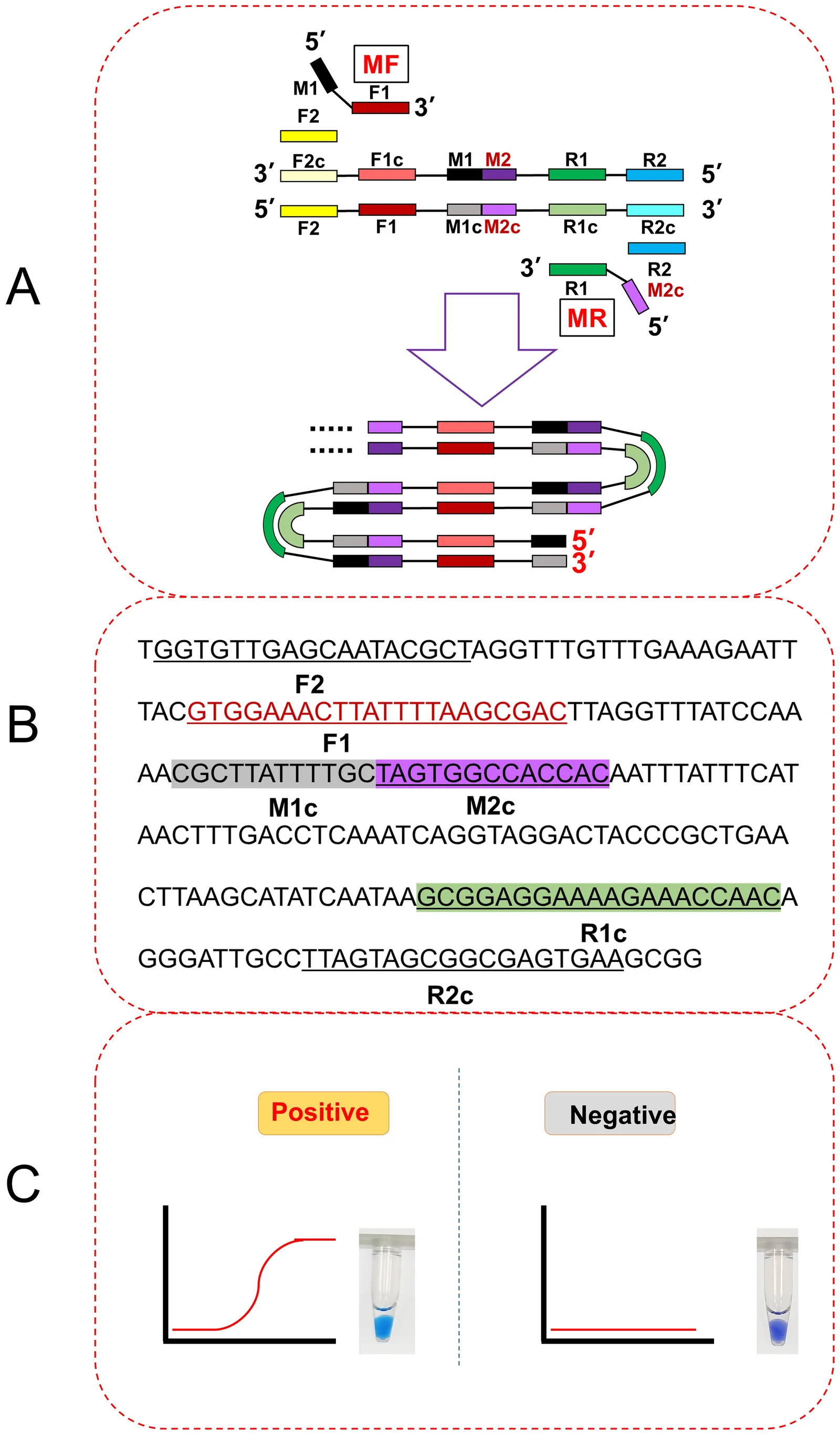
Scheme of the closed dumbbell-mediated isothermal amplification (CDA) method. **(A)** Overall scheme for the primers used in the CDA method. **(B)** Partial sequences of the *Candida tropicalis ITS2* gene used to design the primers for the CDA method. **(C)** Diagram of the CDA assay for *C. tropicalis* detection.

More than four pairs of inner (basic) CDA primers were designed based on the ITS2 region of the *C. tropicalis* genome (accession no. MT149217.1). As depicted in **Figure 1A**, the forward primer (MF) contains sequences complementary to M1 (M1c) and sequences complementary to F1c (F1). The reverse primer (MR) contains sequences (M2) complementary to M2c and R1. The outer primers (F2 and R2) were designed to enhance the amplification efficiency by accelerating the CDA reaction. The binding sites of all *C. tropicalis* CDA primers employed in this study are indicated using different lines or colors (**Figure 1B**). The primer sequences are listed in **Table 1**; **Supplementary Table 1** and sent for chemical synthesis after *in silico* evaluation. The primers used in this research were purchased from BGI Biological Engineering Technology and Services Co. Ltd. (Shenzhen, China).

**Table 1:** *Candida tropicalis* closed dumbbell-mediated isothermal amplification (CDA) primers and PCR primers targeting the *ITS2* gene (MT149217.1).

Target	Method	Primer	Sequence (5′→3′)
*Candida tropicalis ITS2* gene	CDA	CT-MF	GCAAAATAAGCG–GTGGAAACTTATTTTAAGCGAC
		CT-MR	TAGTGGCCACCAC–GTTGGTTTCTTTTCCTCCGC
		CT-F2	GGTGTTGAGCAATACGCT
		CT-R2	TTCACTCGCCGCTACTAA
	PCR	CT-PF	ATGAAGAACGCAGCGAAA
		CT-PR	AAGAGCCAGACCCAAAGATA

### 2.4 CDA assay for *C. tropicalis* detection

The CDA reactions were conducted following the instructions reported by our group (Mao et al., 2022a). Briefly, a CDA reaction was conducted in a PCR tube with 25 μl mixture including 2.5 μl of 10× isothermal amplification buffer and 1 μl *Bst* 2.0 WarmStart DNA polymerase from New England Biolabs (Ipswich, MA, USA), 0.25 μl MF and MR (100 μM each), 1 μl of 20× EvaGreen from Biotium (Hayward, CA, USA) or 3 mM hydroxy naphthol blue (HNB) from Sigma-Aldrich (St. Louis, MO, USA), and nucleic acid samples. Finally, nuclease-free water was used to adjust the volume. Before closing the lid of the reaction tube, the reaction mixture was covered with 30 μl paraffin oil (Sangon, Shanghai, China). The accelerate primers were added by replacing the water (with a final concentration in the reaction mixture of 0.2 μM) to obtain the outer primers that aided the CDA (O-CDA) reaction. The negative control (NC) was prepared using non-targeted DNA sequence nucleic acid samples or simply replaced with nuclease-free water (Sangon, Shanghai, China). The reaction was incubated at 60°C for 60 min and 85°C for 10 min in a CFX96 Real-Time PCR Detection System (Bio-Rad, Hercules, CA, USA) or a Q3 Real-Time PCR instrument (Applied Biosystems, Foster City, CA, USA).

The fluorescent dye EvaGreen was added into the reaction tube for real-time amplification monitoring and final melting curve analysis of the DNA products. HNB was added in the CDA mixture for the validation of the visual on-site detection. Positive reactions were determined by a color change from violet to sky blue, while the color of the negative reactions remained unchanged (**Figure 1C**).

### 2.5 Assessment of the established *C. tropicalis* CDA assay

To evaluate the analytical sensitivity of the developed *C. tropicalis* CDA, its limit of detection (LOD) was assessed using 10-fold serial dilutions prepared from the original extracted *C. tropicalis* DNA stock. The concentrations were as follows: 5.6 × 10^−1^, 5.6 × 10^−2^, 5.6 × 10^−3^, 5.6 × 10^−4^, 5.6 × 10^−5^, 5.6 × 10^−6^, and 5.6 × 10^−7^ ng/μl. The LOD tests of the *C. tropicalis* CDA assay were repeated in triplicate.

To assess the specificity of the developed *C. tropicalis* CDA assay, we performed the test using clinical DNA samples extracted from three other related *Candida* species, i.e., *C. albicans*, *C. glabrata*, and *C. parapsilosis*, and non*-Candida* clinical DNA samples including *P. aeruginosa*, *S. pneumoniae, S. maltophilia, A. fumigatus*, *H. influenzae*, *S. agalactiae*, *E. coli*, *S. sonnei*, *S. aureus*, *V. parahaemolyticus*, and *K. pneumoniae.* The results of the real-time fluorescence monitoring and endpoint visual assessment of the *C. tropicalis* CDA assays were recorded for subsequent analysis of the specificity and sensitivity of the established assay. The online program “Diagnostic test evaluation calculator” (https://www.medcalc.org/calc/diagnostic_test.php) was adopted for statistical analysis of the detection performance of the *C. tropicalis* CDA assay.

### 2.6 Real-time quantitative PCR reaction of *C. tropicalis* detection

The real-time qPCR assay of *C. tropicalis* was performed using primers PF (5′-ATGAAGAACGCAGCGAAA-3′) and PR (5′-AAGAGCCAGACCCAAAGATA -3′) (**Table 1**). The same DNA samples were adopted in the qPCR method to serve as a comparator and in order to validate and confirm the results of the *C. tropicalis* CDA assay. The total volume of 50 μl in the qPCR reaction contained 25 μl of 2× Taq PCR Mix (Sangon, Shanghai, China), 10 mM of each primer, 1× EvaGreen, and DNA sample. The PCR reaction procedures were set as follows: denaturation at 95°C for 2.5 min, annealing at 49°C for 30 s, and extension at 72°C for 30 s, for a total of 35 cycles. Finally, melting curve analysis of the PCR products was conducted on the same instrument following thermal cycling.

## 3 Results

### 3.1 Design and screening of the CDA primers for *C. tropicalis* gene amplification

Following initial *in silico* assessment, four *C. tropicalis* CDA primer pairs were chosen for further experimental validation (**Supplementary Table 1**). The optimal primer set was determined based on the real-time amplification curves and subsequent melting curve analysis of the reaction products obtained from *C. tropicalis* DNA amplification.

**Figure 2:**
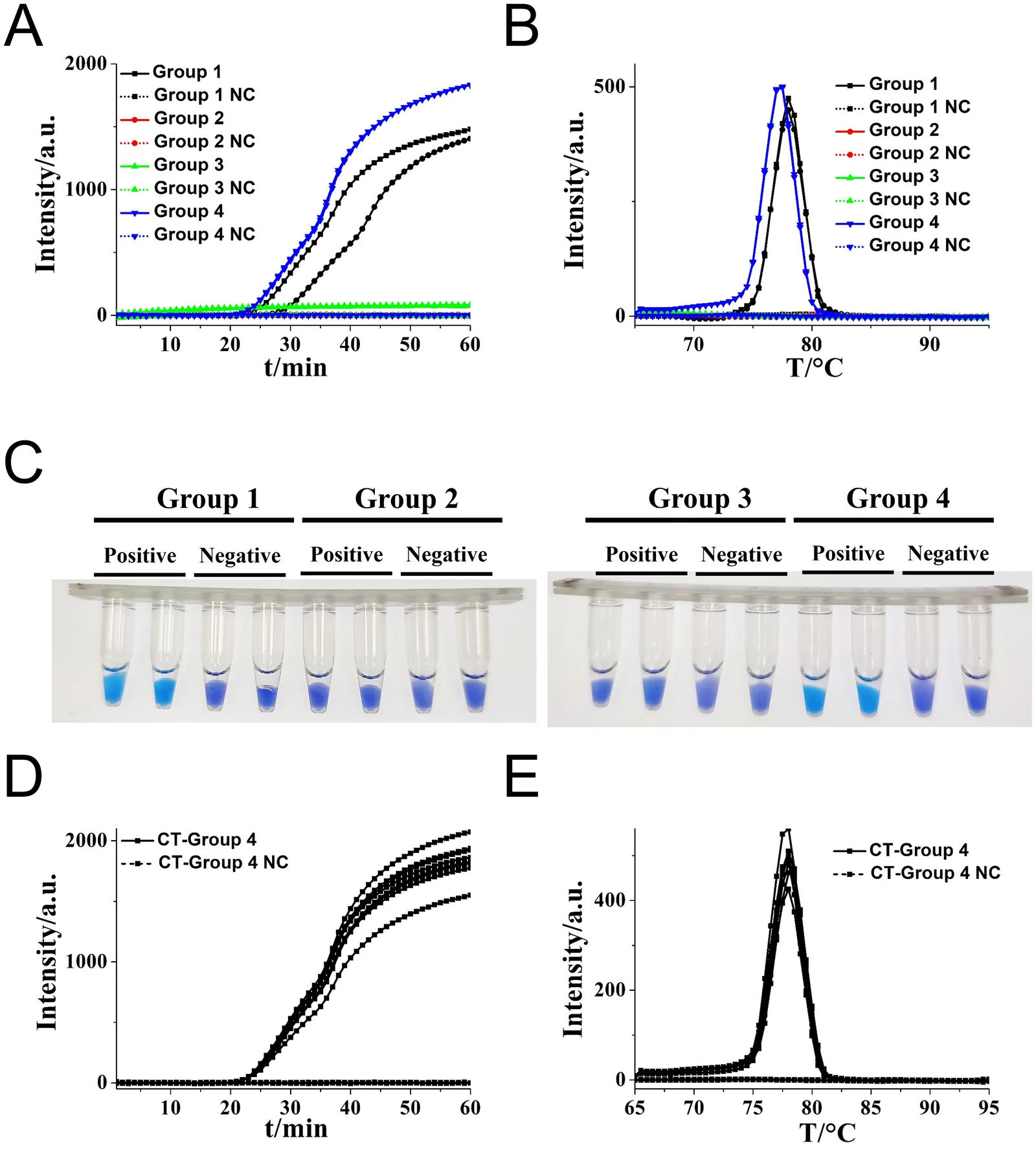
Selection and validation of the inner closed dumbbell-mediated isothermal amplification (CDA) primers of *Candida tropicalis*. **(A)** Real-time fluorescence plots of different CDA primer groups targeting the *C. tropicalis* ITS2 gene. **(B)** Melting curve analysis of the *C. tropicalis* CDA products by real-time PCR. *NC*, negative control. **(C)** Inner primer screening based on colorimetric CDA reaction of *C. tropicalis* DNA (two positive reactions and two negative reactions in each group). **(D)** Real-time fluorescence plot (group 4 primers) for *C. tropicalis* DNA (eight positive reactions and eight negative reactions). **(E)** Melting curve analysis of *C. tropicalis* CDA products by the group 4 CDA primers (eight positive reactions and eight negative reactions).

The amplification efficiency was primarily assessed based on the cycle threshold (*C*_t_) value derived from the *C. tropicalis* CDA amplification curve. Accordingly, the primer pair with the lowest *C*_t_ value was regarded as having the highest preliminary efficiency. Analysis of the amplification curves demonstrated that the fourth primer pair outperformed the other three inner basic primer sets in terms of amplification efficiency of the *C. tropicalis* CDA reactions in **Figure 2A** when identical 5.6 × 10^−2^ ng/μl of the *C. tropicalis* DNA were adopted for each primer group. Specifically, primer 1 demonstrated inconsistent *C*_t_ values between duplicate reactions in the CDA assay, which may have been caused by random hybridization between oligo DNA. Given this level of variability, primer 1 was deemed unsuitable for further optimization. The *C*_t_ value of the fourth group of CDA amplification of 5.6 × 10^−2^ ng/μl of the *C. tropicalis* DNA was 22 min, with a melting temperature (*T*_m_) of the amplification product of 77.50°C. No nonspecific amplification was observed in the NC (**Figures 2A, B**).

Visual endpoint detection further confirmed that the fourth *C. tropicalis* CDA primer set was suitable for on-site detection (**Figure 2C**). In addition, eight positive and eight negative replicates using the fourth *C. tropicalis* CDA primer set were included for subsequent validation. As presented in **Figures 2D, E**, the amplification and melting curve analyses confirmed the high reproducibility and stability of the established *C. tropicalis* CDA assay.

### 3.2 Optimization of the *C. tropicalis* CDA primer set

**Figure 3:**
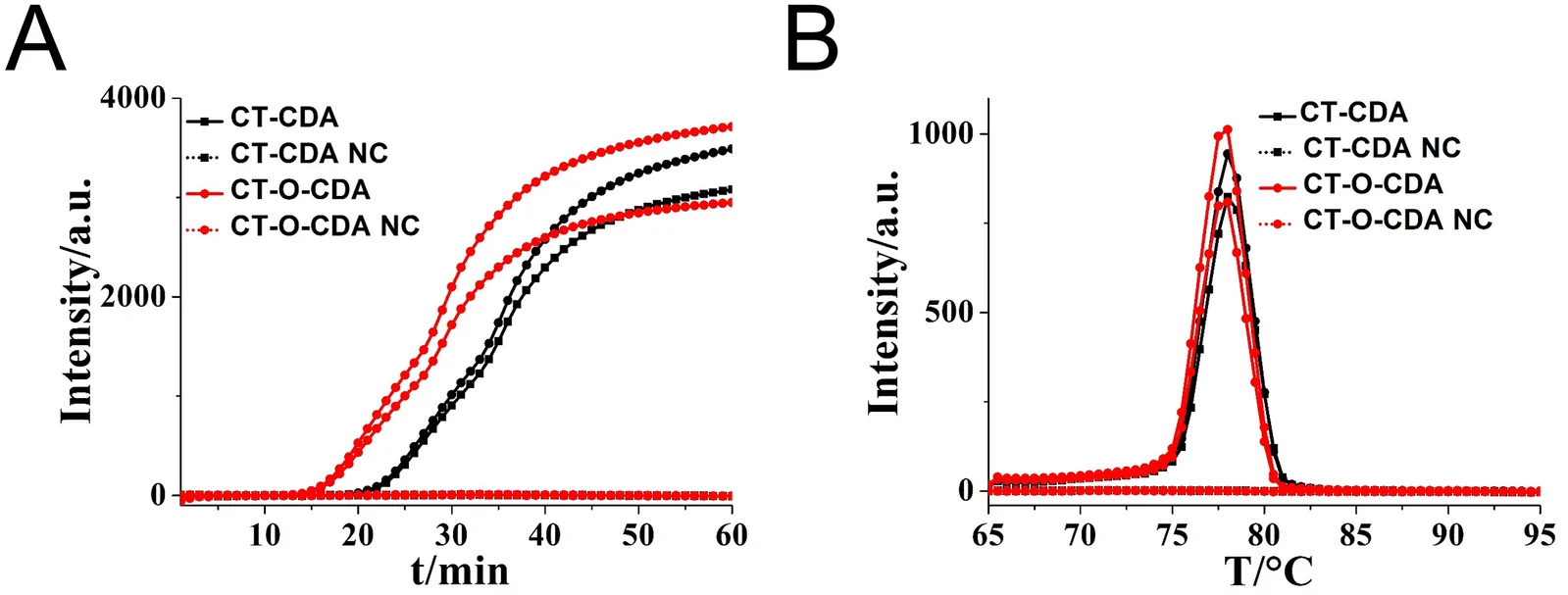
Real-time fluorescent amplification curves of Candida tropicalis DNA by closed dumbbell-mediated isothermal amplification (CT-CDA) and outer primer aided CDA (CT-O-CDA). **(A)** Amplification curves of CT-CDA and the CT-O-CDA assay. **(B)** Melting curve analysis o CT-CDA and the CT-O-CDA assay. PC, positive control; NC, negative control.

Outer primers were designed and validated to enhance the amplification efficiency of the established inner primer-based CDA assay for *C. tropicalis* DNA amplification. As shown in **Figure 3A**, the addition of accelerate (outer) primers led to a substantial improvement in the reaction efficiency of the CDA assay. The *C*_t_ value of the CDA method for the amplification of *C. tropicalis* (5.6 × 10^−2^ ng/μl) decreased to 17 min (a reduction of 5 min).

The results of the real-time amplification curve (**Figure 3A**) and subsequent melting curve analysis (**Figure 3B**) presented good repeatability of the *C. tropicalis* CDA assay. As shown in **Figures 2B** and **3B**, the melting curve analysis of the CDA amplicons revealed consistent patterns. These results confirmed that the inclusion of accelerator primers not only improved the reaction performance but also did not induce nonspecific amplification. Moreover, the melting temperatures of the amplicons shown in **Figures 2B, E** and **3B** were consistent with one another. This finding provides additional evidence for the satisfactory reproducibility of the *C. tropicalis* CDA tests in different batches.

### 3.3 Determination of the limit of detection of the *C. tropicalis* CDA assay

**Figure 4:**
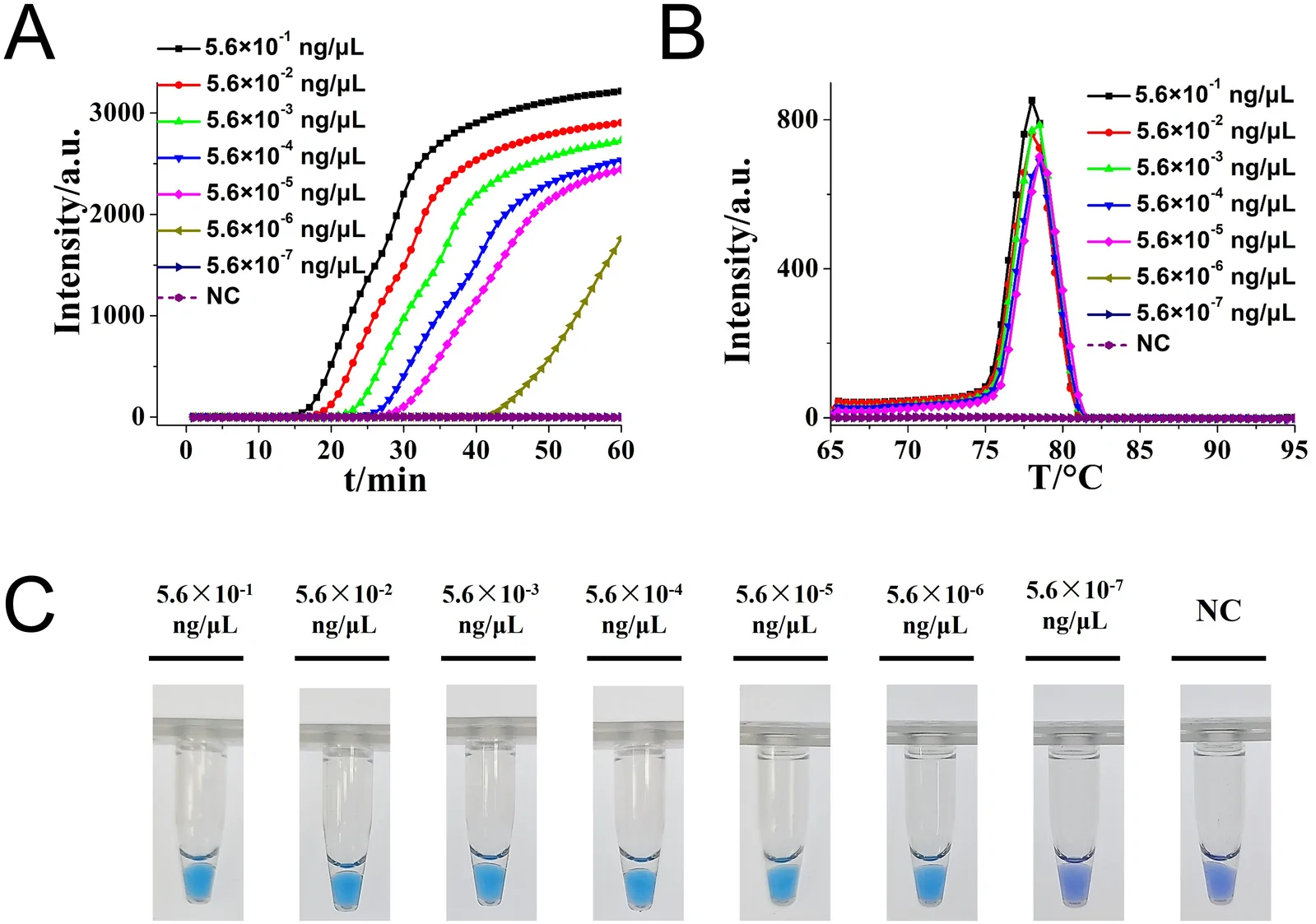
Sensitivity analysis of the *Candida tropicalis* closed dumbbell-mediated isothermal amplification (CDA) method by real-time fluorescent monitoring and endpoint visual assessment. **(A)** *C. tropicalis* CDA amplification was monitored per 1 min with different concentrations of DNA. The reaction was carried out at 60°C for 60 min. **(B)** Melting curve analysis of the *C. tropicalis* CDA products after amplification of different concentrations of extracted DNA. **(C)** Sensitivity analysis of the *C. tropicalis* CDA method for the detection of *C. tropicalis* DNA by visual endpoint monitoring. The DNA concentrations were as follows: 5.6 × 10^−1^, 5.6 × 10^−2^, 5.6 × 10^−3^, 5.6 × 10^−4^, 5.6 × 10^−5^, 5.6 × 10^−6^, and 5.6 × 10^−7^ ng/μL and NC (negative control, i.e., reaction without the targeted template DNA).

**Figure 5:**
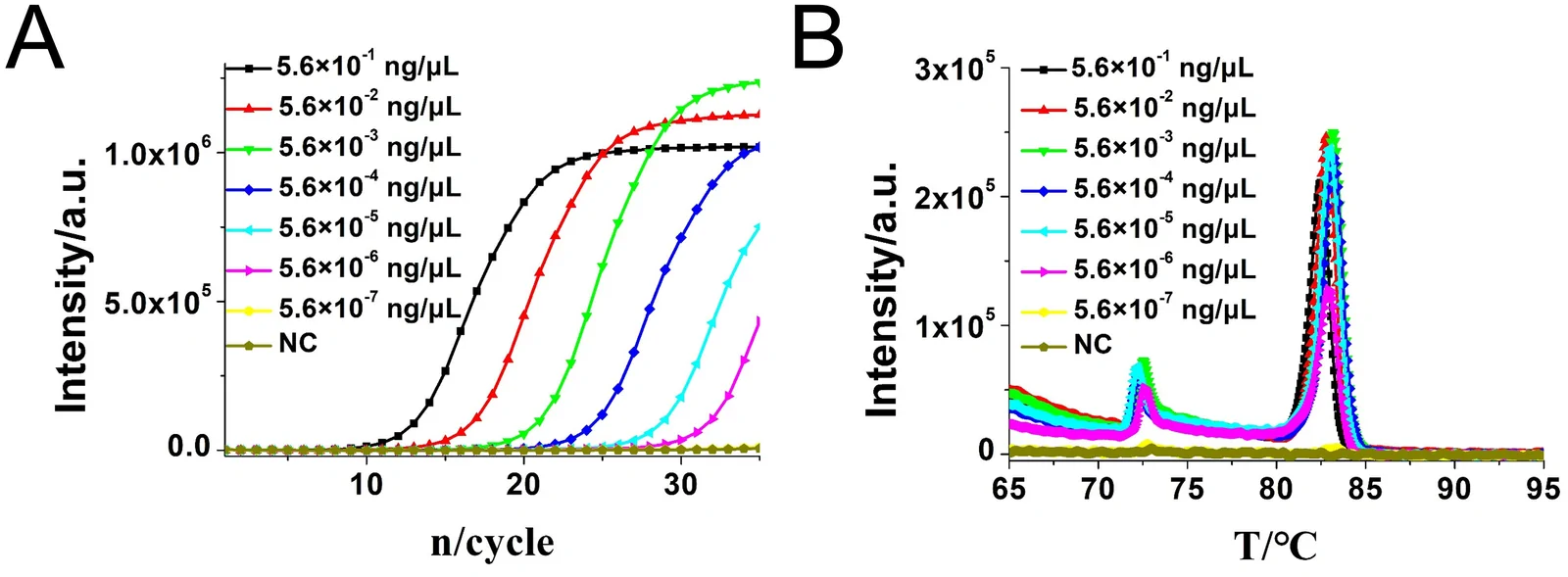
Real-time quantitative PCR assay for *Candida tropicalis* DNA detection. **(A)** Real-time amplification curve. **(B)** Melting curve analysis of *C. tropicalis* by the real-time quantitative PCR assay. NC, negative control reaction without the template.

The *C. tropicalis* CDA assay was established on the basis of preliminary evaluation and optimization of the primer set. Subsequent assessments were performed to further validate the performance of the developed assay. As shown by the real-time fluorescence plot and the color change of the reaction mixture, the minimum detection limit of the CDA assay for *C. tropicalis* was 5.6 × 10^−6^ ng/μl DNA (**Figures 4A, B**), which is similar to that of the qPCR approach (**Figures 5A, B**). The melting curve analysis of the *C. tropicalis* CDA products showed that the *T*_m_ was 77.50°C (**Figure 4B**), which is identical to the aforementioned *T*_m_ in **Figures 2B, E** and **3B**. These results further provided proof that the *C. tropicalis* CDA assay is sensitive and specific for the detection of the targeted DNA sequence.

### 3.4 Assessment of the sensitivity and specificity of the *C. tropicalis* CDA assay

**Figure 6:**
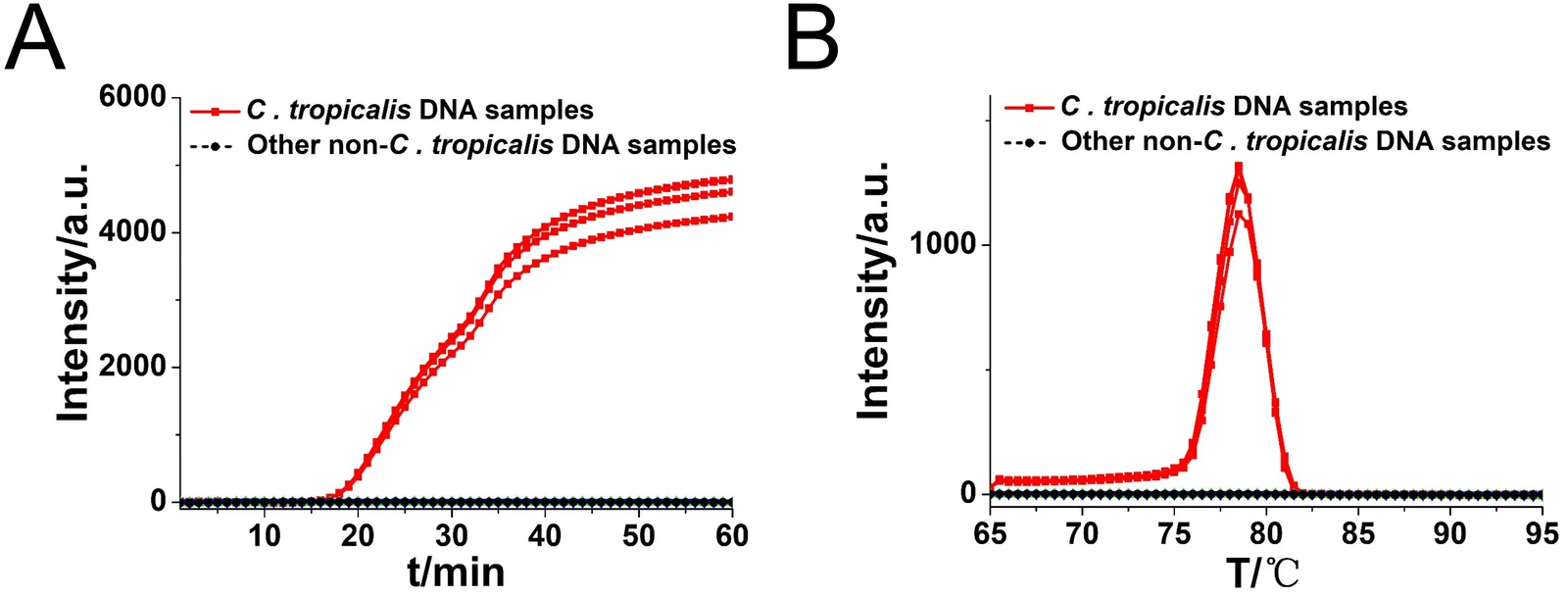
Real-time fluorescent amplification *Candida tropicalis* closed dumbbell-mediated isothermal amplification (CDA) assay. Real-time *C. tropicalis* CDA reactions were carried out at 60°C for 60 min. **(A)** Real-time *C. tropicalis* CDA for the *C. tropicalis* samples and other DNA samples (*Candida glabrata*, *Candida parapsilosis*, *Candida albicans*, *Pseudomonas aeruginosa, Streptococcus pneumoniae, Stenotrophomonas maltophilia*, *Aspergillus fumigatus, Haemophilus influenzae, Streptococcus agalactiae, Escherichia coli, Shigella sonnei, Staphylococcus aureus, Vibrio parahaemolyticus*, and *Klebsiella pneumoniae*). **(B)** Melting curve analysis of the *C. tropicalis* CDA products.

**Figure 7:**
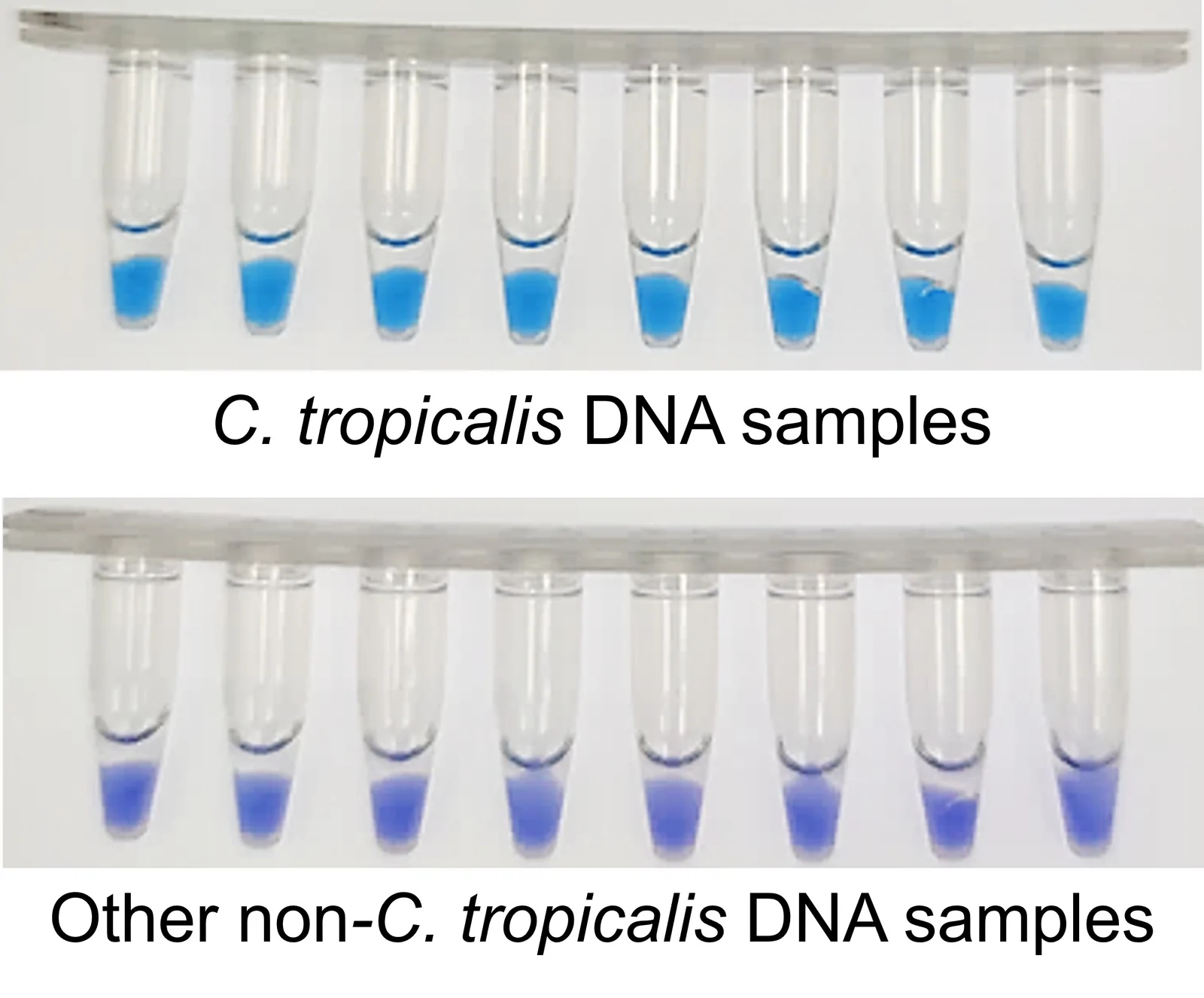
Endpoint colorimetric *Candida tropicalis* method using hydroxy naphthol blue (HNB). Extracted DNA samples from *Candida tropicalis* and other non-*C. tropicalis* mixtures (*Candida albicans*, *Candida glabrata*, *Candida parapsilosis*, *Pseudomonas aeruginosa*, *Streptococcus pneumoniae*, *Stenotrophomonas maltophilia*, *Aspergillus fumigatus*, *Haemophilus influenzae*, *Streptococcus agalactiae*, *Escherichia coli*, *Shigella sonnei*, *Staphylococcus aureus*, *Vibrio parahaemolyticus*, and *Klebsiella pneumoniae*).

To further characterize the analytical sensitivity and specificity of the *C. tropicalis* CDA method, purified DNA from the standard *C. tropicalis* strain was utilized as a positive control. A total of 60 batches of clinical *C. tropicalis* DNA samples were tested. Extracted clinical DNA samples of the phylogenetically related *C. albicans*, *C. glabrata*, and *C. parapsilosis* and 11 non-*Candida* clinical DNA samples were tested using the developed *C. tropicalis* CDA assay. The determined C. *tropicalis* CDA primers demonstrated specific identification and amplification of the intended DNA sequences (**Figure 6A**). Moreover, the melting curve analysis determined the *T*_m_ of *C. tropicalis* CDA amplification products as 77.50°C (**Figure 6B**), and no variation was observed among the positive control and various DNA samples. The *T*_m_ value corroborated previous results from **Figures 2B, E**, **3B**, and **4B**, reflecting the high stability and reproducibility of the *C. tropicalis* CDA assay. In addition, the CDA assay, whether monitored by real-time fluorescence or the endpoint visual readout, enabled accurate differentiation of *C. tropicalis* (**Figures 6**, **7**; **Supplementary Figures 1**, **2**). **Table 2** displays the statistical analysis results of the *C. tropicalis* CDA assay performance in detecting all DNA samples indicated above.

**Table 2:** Determination of the sensitivity and specificity of the *Candida tropicalis* closed dumbbell-mediated isothermal amplification (CDA) assay.

Species	Sample number		Sensitivity	Specificity	Accuracy
	*C. tropicalis* CDA	Defined	95% CI	95% CI	95% CI
*Candida tropicalis*	120	120	1.0 (97.0–100.0)	1.0 (98.5–100.0)	1.0 (99.0–100.0)
*Candida parapsilosis*	0	16			
*Candida glabrata*	0	16			
*Candida albicans*	0	16			
*Pseudomonas aeruginosa*	0	16			
*Streptococcus pneumoniae*	0	16			
*Stenotrophomonas maltophilia*	0	16			
*Aspergillus fumigatus*	0	16			
*Haemophilus influenzae*	0	16			
*Streptococcus agalactiae*	0	16			
*Escherichia coli*	0	16			
*Shigella sonnei*	0	16			
*Staphylococcus aureus*	0	16			
*Vibrio parahaemolyticus*	0	16			
*Klebsiella pneumoniae*	0	16			
Total	120	364			

Statistical analysis was carried out using the online program “Diagnostic test evaluation calculator” (https://www.medcalc.org/calc/diagnostic_test.php). Sensitivity was defined as the proportion of reference-positive subjects correctly identified as positive [TP/(TP + FN)], while specificity was defined as the proportion of reference-negative subjects correctly identified as negative [TN/(FP + TN)].

CI, confidence interval.

## 4 Discussion

It has been reported that *C. tropicalis* ranks as the second most frequent opportunistic fungal pathogen affecting humans, surpassed only by *C. albicans* infections (Yu et al., 2025). Evidence has shown that *C. tropicalis* infection may cause the worst prognosis of sepsis (Ko et al., 2019) and 40% mortality rates (Gunsalus et al., 2016). However, the evolving resistance of *C. tropicalis* to fluconazole is occurring at a much faster rate than the treatment for *C. albicans* infections (Lo et al., 2020). The currently available antifungal drugs have limited efficacy against emerging drug-resistant strains, a growing concern that underscores the urgent need for rapid and accurate *C. tropicalis* detection methods to guide effective clinical management and prevention strategies.

Although the commercialized CHROMagar™ culture method is widely adopted for *Candida* identification, it suffers from two key drawbacks: extended turnaround time and potential false negatives (Kim et al., 2002; Wohlmeister et al., 2017). Matrix-assisted laser desorption/ionization time-of-flight mass spectrometry (MALDI-TOF MS) offers rapid and data-rich identification of yeasts; nevertheless, its widespread adoption is constrained in resource-limited areas by the need for skilled personnel, expensive equipment, and substantial laboratory space (Qian et al., 2008). PCR and DNA sequencing, both recognized as sensitive and specific molecular diagnostic tools, have been applied for the detection of *C. tropicalis* (Wu et al., 2014). Despite their utility, these methods are still cumbersome and poorly suited for adoption in resource-constrained areas where yeast infection diagnostics are needed (Wilner et al., 2023). In addition, the well-established LAMP has been applied for the detection of a variety of yeasts, including *C. albicans* and *C. tropicalis* (Kasahara et al., 2014). However, LAMP has exhibited flaws such as a complicated primer design and high rates of nonspecific amplification (Gao et al., 2019; Schneider et al., 2019; Lamas et al., 2023).

The *C. tropicalis* CDA assay established in this work exhibited excellent analytical performance, including 100% sensitivity (95%CI = 97.0%–100%) and 100% specificity (95%CI = 98.5%–100%), high accuracy, and a low LOD (**Figure 4**, **Table 2**). The preliminary LOD was initially estimated using three replicate measurements at each of the tested concentration levels as part of an exploratory dose–response screening. The developed CDA assay proved simpler and more efficient than PCR-based methods for the detection of *C. tropicalis*. The experimental results demonstrated that the lower LOD of the *C. tropicalis* CDA assay was 5.6 × 10^−6^ ng/μl of the extracted *C. tropicalis* DNA (approximately 10 copies/μl), which is similar to that of qPCR (**Figures 4**, **5**).

In the evaluation of the specificity index of the *C. tropicalis* CDA assay (**Figures 6**, **7**), no signal of reaction was found in the amplification assays of the DNA samples extracted from the closely related *C. glabrata*, *C. parapsilosis*, and *C. albicans*. In addition, no cross-reactivity was observed: the *C. tropicalis* CDA assay produced no amplification signals when challenged with 11 phylogenetically distant pathogens: *P. aeruginosa*, *S. pneumoniae*, *S. maltophilia*, *A. fumigatus*, *H. influenzae*, *S. agalactiae*, *E. coli*, *S. sonnei*, *S. aureus*, *V. parahaemolyticus*, and *K. pneumoniae.* The color change of the reaction mixture was consistent with the real-time fluorescent curve when the *C. tropicalis* CDA assay was applied to detect the same DNA samples, confirming the suitability of the assay for on-site detection.

Several limitations of this study should be acknowledged. Firstly, all clinical samples were collected from a single center over a relatively short enrollment period of 5 months. This restricted geographic and temporal sampling may have limited the generalizability of our findings to other populations or seasons, particularly given the potential for regional variations in pathogen genotypes and seasonal fluctuations in disease prevalence. However, it should be noted that our center is a tertiary referral hospital serving a large and diverse patient population from both urban and rural areas across Zhejiang Province, which partially mitigates the concern of demographic homogeneity. Moreover, external validation using multicenter cohorts and longitudinal sampling across different seasons is currently underway and will be essential to confirm the robustness of the diagnostic performance estimates reported herein. Secondly, the present study evaluated the performance of our assay exclusively on purified DNA extracted from clinical specimens. The diagnostic performance estimates reported herein should be interpreted as the intrinsic capability of the assay rather than its real-world clinical utility at the POC level. We deliberately chose this initial validation strategy to establish the proof-of-concept of CDA detection. Ongoing work in our laboratory is now focused on adapting this assay for direct testing of crude specimens. In addition, we explain that this was a deliberate design choice for this proof-of-concept phase, as MALDI-TOF MS is a phenotypic/proteomic method whereas our assay is genotypic, and that a direct comparison would address clinical workflow equivalence rather than analytical accuracy—which we considered as a separate validation phase.

Overall, the total detection time for the *C. tropicalis* CDA assay was 60 min, substantially shorter than the 120 min required by conventional PCR. When evaluated against qPCR and NGS for *C. tropicalis* detection, the developed *C. tropicalis* CDA assay demonstrated apparent 100% sensitivity (95%CI = 97.0%–100%), specificity (95%CI = 98.5%–100%), and accuracy (95%CI = 99.0%–100%) (**Table 2**). We explicitly state that our findings represent a proof of concept rather than a guarantee of perfect performance in larger cohorts. In the specificity and sensitivity validation, the *C. tropicalis* CDA assay was applied to 120 batches of *C. tropicalis* DNA samples (standard and clinical) and 244 batches of target-free clinical DNA samples, thereby supporting its clinical utility. Moreover, the results of the real-time amplification curves, melting curve, and endpoint color assessment of the *C. tropicalis* CDA assay were consistent, showed the excellent stability and reproducibility of the assay, and laid the foundation for its future application.

## 5 Conclusions

In this work, we developed a *C. tropicalis* CDA assay that enables rapid, sensitive, and specific DNA detection via either real-time amplification or visual colorimetric endpoints. This method holds potential to streamline *C. tropicalis* diagnosis and inform effective clinical management of infections caused by this pathogen. When implemented in fluorescence-based microfluidic platforms, the real-time amplification curve-based *C. tropicalis* CDA assay demonstrates a markedly improved efficiency (Lehnert and Gijs, 2024). With its endpoint visual readout, the *C. tropicalis* CDA assay can be performed in a standard water bath, offering a future approaching the development of POC detection in underdeveloped regions (Zhang et al., 2022).

## Data Availability

The original contributions presented in the study are included in the article/**Supplementary Material**. Further inquiries can be directed to the corresponding author/s.
