# Supplementary material for: Sensitive and rapid detection of Candida tropicalis using closed dumbbell-mediated isothermal amplification
Source: Front Cell Infect Microbiol. 2026 Aug 21;16:1875246. doi: 10.3389/fcimb.2026.1875246 (PMC13541524; doi:10.3389/fcimb.2026.1875246)
Supplement: Supplementary file 1 [file Table1.docx]

Supplementary Material

Rapid and sensitive detection of *Candida tropicalis* using closed dumbbell mediated isothermal amplification (CDA)

**Yangbo Fu^1^**^,^ **^+^,** **Jing Shen^1^**^,^ **^+^, Haizhong Jiang^1^, Yanli Zhang ^1^, Xuhan Chen ^2^**^,^ **^3^, and Rui Mao ^2, 3, *^**

^1^ The First Affiliated Hospital of Ningbo University, Ningbo 315000, Zhejiang, China

^2^ Ningbo NO. 2 Hospital, Wenzhou Medical University, Ningbo 315010, Zhejiang, China.

^3^ Ningbo Institute of Life and Health Industry, University of Chinese Academy of Sciences, Ningbo 315010, China.

^+^ These authors contributed equally to this work

*** Correspondence:**

Rui Mao

[mr3749@163.com](mailto:mr3749@163.com)

# 1 Supplementary Figures and Tables

## 1.1 Supplementary Table

###### Supplementary Table 1 Four pairs of CT-CDA primers were designed by DNAMAN

| **Target** | **Method** | **Primer** | **Sequence (5´→3´)** |
| --- | --- | --- | --- |
| ITS2 | CDA | CT-MF-1 | GCATGCCCTTGATGAAGAACGCAGCGA |
|  |  | CT-MR-1 | CTGTTTGAGCTGCTCAACACCAAACCC |
|  |  | CT-MF-2 | TCAAAGATTCGCTCTTGGTTCTCGCATC |
|  |  | CT-MR-2 | ACGCACATTGAATGACGCTCAAACAGG |
|  |  | CT-MF-3 | AGATTCGATGCTCTTGGTTCTCGCATC |
|  |  | CT-MR-3 | TTGAACGCACAATGACGCTCAAACAGG |
|  |  | CT-MF-4 | GCAAAATAAGCGGTGGAAACTTATTTTAAGCGAC |
|  |  | CT-MR-4 | TAGTGGCCACCACGTTGGTTTCTTTTCCTCCGC |

## 1.2 Supplementary **Figure**

**Supplementary Figure 1**

Real time fluorescent amplification curve monitored by Eva green. 1. Standard *Candida tropicalis* DNA; 3, 5, 7, extracted DNA samples from *Candida tropicalis*; 2, 4, 6, 8, other non-*Candida tropicalis* (*Candida albicans, Candida glabrata, Candida parapsilosis, Pseudomonas aeruginosa, Streptococcus pneumoniae, Stenotrophomonas maltophilia, Aspergillus fumigatus, Haemophilus influenzae, Streptococcus agalactiae, Escherichia coli, Shigella sonnei, Staphylococcus aureus, Vibrio Parahaemolyticus and Klebsiella pneumoniae*).

**Supplementary Figure 2**

Endpoint colorimetric *C. tropicalis* assay monitored by HNB. 1. Standard *Candida tropicalis* DNA; 3, 5, 7, extracted DNA samples from *Candida tropicalis*; 2, 4, 6, 8, other non-*Candida tropicalis* (*Candida albicans, Candida glabrata, Candida parapsilosis, Pseudomonas aeruginosa, Streptococcus pneumoniae, Stenotrophomonas maltophilia, Aspergillus fumigatus, Haemophilus influenzae, Streptococcus agalactiae, Escherichia coli, Shigella sonnei, Staphylococcus aureus, Vibrio Parahaemolyticus and Klebsiella pneumoniae*).
